# Comparison of Postoperative Neck Pain and Discomfort, Swallowing Difficulty, and Voice Change After Conventional Open, Endoscopic, and Robotic Thyroidectomy: A Single-Center Cohort Study

**DOI:** 10.3389/fendo.2018.00416

**Published:** 2018-07-19

**Authors:** Tae Kwun Ha, Dong Wook Kim, Ha Kyoung Park, Gi Won Shin, Young Jin Heo, Jin Wook Baek, Yoo Jin Lee, Hye Jung Choo, Do Hun Kim, Soo Jin Jung, Ji Sun Park, Sung Ho Moon, Ki Jung Ahn, Hye Jin Baek, Taewoo Kang

**Affiliations:** ^1^Department of General Surgery, Busan Paik Hospital, Inje University College of Medicine, Busan, South Korea; ^2^Department of Radiology, Busan Paik Hospital, Inje University College of Medicine, Busan, South Korea; ^3^Department of Otorhinolaryngology-Head and Neck Surgery, Busan Paik Hospital, Inje University College of Medicine, Busan, South Korea; ^4^Department of Pathology, Busan Paik Hospital, Inje University College of Medicine, Busan, South Korea; ^5^Department of Nuclear Medicine, Busan Paik Hospital, Inje University College of Medicine, Busan, South Korea; ^6^Department of Anesthesiology and Pain Medicine, Busan Paik Hospital, Inje University College of Medicine, Busan, South Korea; ^7^Department of Radiation Oncology, Busan Paik Hospital, Inje University College of Medicine, Busan, South Korea; ^8^Department of Radiology, Gyeongsang National University School of Medicine and Gyeongsang National University Changwon Hospital, Changwon, South Korea; ^9^Busan Cancer Center, Department of Surgery, Pusan National University Hospital, College of Medicine, Pusan National University, Busan, South Korea

**Keywords:** thyroid, surgery, robotic, endoscopic, conventional, complication

## Abstract

**Background:** The objective of this study was to compare the postoperative neck pain and discomfort, swallowing difficulty, and voice change after conventional open thyroidectomy (COT), endoscopic thyroidectomy (ET), or robotic thyroidectomy (RT) performed by a single surgeon.

**Methods**: From January 2013 to December 2017, 254 patients underwent COT, ET, or RT performed by a single surgeon and completed a postoperative symptom survey conducted in the outpatient clinic by three nurses. The survey collected information on postoperative neck pain and discomfort, swallowing difficulty, and voice change.

**Results:** Of the 254 patients, 169 underwent COT, 32 underwent ET, and 53 underwent RT. The mean age in the COT, ET, and RT groups was 50.1, 44.5, and 41.6 years, respectively. The mean interval between thyroidectomy and survey in the COT, ET, and RT groups was 42.7, 50.2, and 9.2 months, respectively. Postoperative neck pain was significantly higher in the ET and RT groups than in the COT group (*p* = 0.026). The average neck impairment index score in the RT group was significantly higher than that in the COT group (*p* < 0.001). There were no significant differences in pain scale scores, swallowing difficulty, swallowing impairment index, voice change, and voice hand index among the three groups.

**Conclusions:** There were no significant differences in postoperative voice change or swallowing difficulty among the COT, ET, and RT groups, whereas neck pain and discomfort were more common after ET and RT than COT.

## Introduction

Until recently, the gold standard of surgical treatment for thyroid diseases with low morbidity and negligible mortality was conventional open thyroidectomy (COT) through a Kocher collar incision (1, 2). The oncologic characteristics of thyroid cancer, such as slow growth, low recurrence, and good prognosis, led patients to pay more attention to quality of life outcomes, including cosmetic problems and postoperative functional recovery (3). Various surgical methods using endoscopic or robotic systems have been introduced for use in thyroidectomy, and they showed significantly superior aesthetic outcomes compared to COT (4). While endoscopic thyroidectomy (ET) was initially limited to solitary, relatively small, benign, or non-functional tumors, as a result of accumulated surgical experience, ET is now commonly performed for low risk thyroid cancer. However, the two-dimensional visualization, narrow working space in the cervical area, and the rigidity of the instruments makes it difficult to employ ET in delicate operations and central neck dissection (5). Robotic thyroidectomy (RT) was introduced to overcome the limitations of ET. RT is performed using the da Vinci robot that is capable of three-dimensional visualization, hand-tremor stabilization, and fine control with no neck scarring (6).

The excellent aesthetic outcomes and the surgical usefulness of ET and RT in comparison to COT have been previously reported (7–9). Recently, one prospective study investigated postoperative pain after COT and RT (10). Numerous studies have compared pain, voice change, and swallowing difficulty after COT, ET, or RT. However, to the best of our knowledge, none of them have assessed the three types of thyroidectomy when performed by a single surgeon. Thus, the purpose of this study was to identify whether these three approaches have different functional outcomes by administering a survey to patients who underwent thyroidectomy for treatment of benign or malignant diseases by a single surgeon.

## Materials and methods

### Study population

From January 2013 to December 2017, 725 patients (613 women and 112 men; age range, 14–86 years; mean age, 46.9 ± 12.3 years) underwent COT, ET, or RT for thyroid diseases at Busan Pak Hospital performed by a single surgeon (TKH). This surgeon has 8 years' experience in COT (50–80 cases/year), 6 years' experience in ET (20–30 cases/year), and 2 years' experience in RT (40–50 cases/year). Of the 725 patients, 153 were not followed-up postoperatively and 418 did not take this survey. Ultimately, 254 patients (229 women and 25 men; age range, 19–75 years; mean age, 47.6 ± 11.27 years) were included in this study. The institutional review board approved this study, and the need for informed consent was waived.

### Thyroid surgery

In the case of benign neoplasms where potential malignancy could not be excluded, unilateral lobectomy was performed. ET or RT was performed in patients with a malignant neoplasm smaller than 2 cm in size and limited to the thyroid gland regardless of patient age. None of these patients showed either lateral lymph node metastasis or extrathyroidal extension on preoperative imaging studies, distant metastasis, or a history of neck surgery or radiation treatment.

#### COT

The patient was placed in a supine position with the neck extended. A 5 to 6 cm circular incision was performed along the natural crease two finger breadths above the sternal notch, up to both lateral surfaces of the sternocleidomastoid muscle. A subplatysmal flap was created until the deep cervical fascia was exposed. After dividing the strap muscle in the center and inducing its lateral traction, the thyroid was exposed and thyroidectomy was performed.

#### ET (gasless, transaxillary approach)

After a 4 to 6 cm vertical incision was made in the axilla, a subplatysmal skin flap was created from the axilla to the anterior neck through the upper pectoralis major muscle using electrocautery under gross observation. The strap muscle was lifted by approaching the space between the clavicular head and the sternal head of the sternocleidomastoid muscle and the thyroid was subsequently exposed. After creating a sufficient space up to the upper and lower poles of the thyroid gland and the opposite lobe was revealed, Jung's retractor was inserted and the flap was lifted. Through the axillary incision, the operative field was lit by a 30-degree endoscopic camera pointed toward the head. Thyroidectomy was performed using an endoscopic dissector and a grasper that could hold and retract the thyroid gland, as well as a Harmonic Scalpel (Johnson & Johnson Medical, Cincinnati, OH) capable of simultaneous coagulation and cutting.

#### RT (gasless, transaxillary approach)

RT (da Vinci Robot System; Intuitive Surgical, Inc., Sunnyvale, CA) using a gasless transaxillary approach was performed in identical fashion to ET, except for the robot docking procedure, until an operative space was created and an external retractor inserted. Moreover, its key maneuvers were the same as those of COT, except for docking by creating a flap. The patient under general anesthesia was placed in a supine position with the neck extended. The patient's upper arm on the affected side was then lifted and fixed. After the retractor was inserted and the operative field was formed, robot docking was performed. Four robot arms were used. A dual-channel 30-degree endoscope (Intuitive Surgical, Inc.) was placed in the central camera arm using a 12-mm trocar. Harmonic cured shears (Intuitive Surgical, Inc.) were placed on the right side of the scope using an 8-mm trocar, and a Maryland dissector & ProGrasp forceps (Intuitive Surgical, Inc.) were placed at the top and bottom on the left side of the scope. Using these tools, thyroid gland traction and dissection were performed. Thyroidectomy was performed in a manner similar to COT.

### Evaluation of postoperative symptoms

From July 2017 to December 2017, the survey for postoperative symptoms was conducted by three nurses in the outpatient clinic. In each patient, all postoperative symptoms were investigated on the basis of comparison with their preoperative condition. All the patients answered questionnaires regarding the pain score of the surgical area, voice handicap index (VHI), swallowing impairment score (SIS), and neck impairment index (NII) (11–13). The intensity of pain was quantified using the numerical rating scale and divided into mild (1–4), moderate (5–6), and severe (7–10) categories (Table 1). In addition, the NII was scored depending on the existence of discomfort and dysfunction in the neck and shoulder after thyroid surgery, and individual items from the 10-question NII were scored from 1 to 5, with 5 representing higher quality of life responses. The total NII score was scaled to a 100-point cumulative score. Swallowing difficulty was evaluated using the SIS on scale from 0 (no swallowing alteration) to 24 points (most severe swallowing impairment). Non-voice throat symptoms related to ET, including cough, choking, and throat clearing, were also evaluated using these scores. Voice change was assessed using the VHI, which consists of 10 questions with answers ranging from 0 (no voice alteration) to 40 (most severe voice impairment). In the case of voice change, the recurrent laryngeal nerve function was tested using indirect laryngoscopy 1, 3, and 6 months after surgery until the voice recovered. Indirect laryngoscopy was performed in all patients with voice change and transient vocal cord palsy was diagnosed when the palsy lasted for <6 months.

**Table 1:** Survey questions of the neck impairment index, swallowing impairment index, and voice handicap index.

**Statement**	**Score**
**NECK IMPAIRMENT INDEX**	
Are you bothered by neck pain or discomfort?	0 1 2 3 4
Are you bothered by neck stiffness	0 1 2 3 4
Are you bothered by difficulty with self-care activities because of your neck (for example, combining hair, dressing, bathing, etc)?	0 1 2 3 4
Have you been limited in your ability to lift light objects because of your neck?	0 1 2 3 4
Have you been limited in your ability to lift heavy objects because of your neck	0 1 2 3 4
Have you been limited in your ability to reach above for objects because of your neck (for example, for shelves, tables, or counters)?	0 1 2 3 4
Are you bothered by your overall activity level because of your neck?	0 1 2 3 4
Has the treatment of your neck affected your participation in social activities?	0 1 2 3 4
Have you been limited in your ability to do leisure or recreational activities because of your neck?	0 1 2 3 4
Have you been limited in your ability to do work (including work at home) because of your neck?	0 1 2 3 4
0 (not at all), 1 (a little bit), 2 (a moderate amount), 3 (quite a bit), 4 (a lot)	
**SWALLOWING IMPAIRMENT INDEX**	
It requires great effort to swallow	0 1 2 3 4
I feel a throat obstacle during swallowing	0 1 2 3 4
I feel pharyngeal annoyance during bolus transit	0 1 2 3 4
I cough during bolus transit	0 1 2 3 4
I feel a sensation of a foreign body in my pharynx	0 1 2 3 4
I have some difficulties swallowing fluids	0 1 2 3 4
0 (never), 1 (almost never), 2 (sometimes), 3 (almost always), 4 (always)	
**VOICE HANDICAP INDEX**	
My voice makes it difficult for people to hear me	0 1 2 3 4
People have difficulty understanding me in a noisy room	0 1 2 3 4
My voice difficulties restrict my personal and social life	0 1 2 3 4
I feel left out of conversations because of my voice	0 1 2 3 4
My voice makes me feel handicapped	0 1 2 3 4
My voice problem causes me to lose income	0 1 2 3 4
I feel as though I have to strain to vocalize	0 1 2 3 4
The clarity of my voice is unpredictable	0 1 2 3 4
My voice problem upsets me	0 1 2 3 4
People ask, “What's wrong with your voice?”	0 1 2 3 4
0 (never), 1 (almost never), 2 (sometimes), 3 (almost always), 4 (always).	

### Statistical analysis

The data were tested for normal distribution using a Kolmogorov-Smirnov test. Normally distributed variables were compared using the one-way analysis of variance and expressed as the mean ± standard deviation. Tukey's honestly significant difference test was used for the post hoc multiple comparisons. Group comparisons of categorical variables were performed using the χ^2^ test or, for small cell values, Fisher's exact test. All statistical analyses were performed with statistical software (SPSS version 24.0, SPSS Inc, Chicago, IL; and MedCalc version 14.10, MedCalc Software, Ostend, Belgium) and *p* < 0.05 was considered statistically significant.

## Results

Of the 254 patients (female: male = 229:15), 169 underwent COT, 32 underwent ET, and 53 underwent RT. Patient demographics and characteristics are summarized in Table 2. The patient age, sex, and type of thyroid surgery showed a significant difference among the COT, ET, and RT groups (*p* < 0.0001). The mean age in the COT group was significantly higher than in the ET or RT groups. However, there was no statistical difference in patient age between the ET and RT groups. Female predominance was observed in the COT group, while all patients in both the ET and RT groups were female (32:0, 53:0; *p* < 0.0001). As for the type of thyroid surgery, only 41.4% (70/169) of patients in the COT group underwent hemithyroidectomy, whereas the majority in the ET and RT groups underwent hemithyroidectomy. Of those who received surgery for thyroid disease, thyroid cancer comprised the overwhelmingly largest proportion with 243 cases (95.7%), followed by 8 cases of nodular hyperplasia, 3 cases of follicular adenomas, and 1 case of Hurthle cell adenoma. The average interval between the surgery and survey was 36.6 ± 29.1 months (range, 1–82 months). The interval between thyroid surgery and survey in the RT group was shorter than that in the COT and ET groups (*p* < 0.0001).

**Table 2:** Demographics and characteristics of the 254 patients.

**Method Item**	**Conventional (*n* = 169)**	**Endoscopic (*n* = 32)**	**Robot (*n* = 53)**	***P*-value**
Age (mean ± SD, years)	50.1 ± 10.9	44.5 ± 9.3	41.6 ± 10.7	<0.0001
Sex (female: male)	144:25	32:0	53:0	<0.0001
Type of thyroid surgery				<0.0001
Total thyroidectomy	99 (58.6)	2 (6.3)	5 (9.4)	
Hemithyroidectomy	70 (41.4)	30 (93.8)	46 (86.8)	
Isthmusectomy	0	0	2 (3.8)	
Histopathological results				0.606
Papillary thyroid carcinoma	157 (92.9)	31 (96.9)	50 (94.3)	
Follicular thyroid carcinoma	1 (0.6)	1 (3.1)	0	
Medullary thyroid carcinoma	1 (0.6)	0	0	
Poorly differentiated carcinoma	1 (0.6)	0	0	
Hurthle cell adenoma	1 (0.6)	0	0	
Follicular adenoma	1 (0.6)	0	2 (3.8)	
Nodular hyperplasia	7 (4.1)	0	1 (1.9)	
Interval between surgery and survey (mean ± SD, months)	42.7 ± 30.2	50.2 ± 16.9	9.2 ± 4.7	<0.0001

*Data presented in parentheses are percentage of each item. SD, standard deviation*.

In all patients, the survey for postoperative symptoms was performed in a single session after thyroidectomy. The postoperative symptoms after COT, ET, and RT are compared in Table 3. There were no significant differences in the pain scale scores, dysphagia, SIS score, voice change, and VHI among the three groups. However, the ET and RT groups exhibited a higher frequency of swallowing difficulty and higher SIS than the COT group. In terms of voice change and VHI, ET led to poorer outcomes than COT and RT. Of the 254 patients, 1 patient in the COT group exhibited no improvement in voice change. Postoperative neck pain was significantly higher in the ET and RT groups than in the COT group (*p* = 0.026), whereas no significant difference was found in pain scale scores among the three groups (*p* = 0.2). Neck discomfort exhibited a correspondence with NII in the COT, ET, and RT groups. The RT group exhibited the highest frequency of neck discomfort and the highest mean NII, whereas the COT group had the lowest frequency of neck discomfort and lowest mean NII. In all patients with voice change, no direct recurrent laryngeal nerve injury was confirmed through laryngoscopy and complete recovery of the voice was observed.

**Table 3:** Comparison of postoperative symptoms according to the surgical approach in 254 patients.

**Method Complication**	**Conventional (*n* = 169)**	**Endoscopic (*n* = 32)**	**Robot (*n* = 53)**	***P* value**
Neck pain				0.026
Absence	96 (56.8)	13 (40.6)	20 (37.7)	
Presence	73 (43.2)	19 (59.4)	33 (62.3)	
Pain scale (1-10)				0.2
1	96 (56.8)	13 (40.6)	20 (37.7)	
2	27 (16)	8 (25)	7 (13.2)	
3	29 (17.2)	8 (25)	18 (34)	
4	6 (3.6)	1 (3.1)	2 (3.8)	
5	4 (2.4)	0	3 (5.7)	
6	3 (1.8)	0	1 (1.9)	
7	2 (1.2)	1 (3.1)	0	
8	1 (0.6)	0	0	
9	1 (0.6)	0	0	
10	0	1 (3.1)	2 (3.8)	
Neck discomfort				0.001
Absence	109 (64.5)	18 (56.3)	19 (35.9)	
Presence	60 (35.5)	14 (43.8)	34 (64.2)	
Neck impairment index (0–100)	14 ± 19.5	17.3 ± 20.2	23.4 ± 19.5	0.01
Swallowing difficulty				0.626
Absence	106 (62.7)	18 (56.3)	30 (56.6)	
Presence	63 (37.3)	14 (43.8)	23 (43.4)	
Swallowing impairment score 10 (0–24)	2.1 ± 3.6	2.5 ± 3.4	3.4 ± 5.2	0.115
Voice change				0.258
Absence	122 (72.2)	19 (59.4)	40 (75.5)	
Presence	47 (27.8)	13 (40.6)	13 (24.5)	
Voice handicap index 10 (0-?)	1.7 ± 4.2	2.8 ± 5.1	2.1 ± 5.3	0.436
Recovery period (months)	0.8 ± 2.2	1.4 ± 3.1	0.6 ± 1.5	0.228

*Data presented in parentheses are percentage of each item. US, ultrasonography; PTC, papillary thyroid carcinoma. Of the 138 cases, nodule vascularity was analyzed in 135 cases*.

## Discussion

As surgeons have accumulated knowledge of endoscopic head and neck anatomy, various methods of thyroidectomy have been developed for thyroid tumors (2, 4–9). ET and RT have advantages in terms of minimizing the postoperative scar (4–10). However, they are associated with disadvantages such as long operation times, limited operative space, and narrow surgical view in comparison with COT. With the increasing use of ET and RT, evaluation of postoperative symptoms is important for both patients and surgeons. Several studies have reported pain and discomfort of the neck and anterior chest after thyroidectomy (10, 14–16). Postoperative neck pain results from the need to gain exposure for thyroidectomy, retractor installation during the operation, and dissection to secure sufficient space at the opposite side to the axilla and thyroid lesion (16). Recently, one prospective study demonstrated that postoperative pain after RT was equivalent to that after COT (10). In our study, postoperative neck pain was significantly higher in the ET or RT groups than in the COT group, although there was no significant difference in the pain scale scores. The reason for this difference is unclear. However, it may be associated with the fact that the extent of surgical injury in ET or RT is not less than that in COT. In the present study, the incidence of postoperative neck discomfort was higher in the RT group than in the ET or COT groups. After thyroidectomy, the mean NII was significantly higher in the RT group than in the ET or COT groups. The reason for this may be associated with the relatively short experience of a surgeon for RT and the characteristics of RT, such as the longer operation time, wider flap, or larger dissection extent. For clarity, a further study may be required.

Swallowing difficulty is one of the important complications after thyroidectomy. The cause is still unclear, but it is usually transient and occurs early after the surgical procedure (17). The causes may include orotracheal intubation, surgical manipulation, scarring and adhesions of the skin flap to the superficial cervical fascia, and psychological reaction to the surgery (17). In a previous study, the COT group had significantly worse mean SIS scores at both 1 week and 3 months after thyroidectomy (16). However, other investigators reported that there were no differences in swallowing impairment between COT and RT at up to 6 months (18). In our study, no significant difference was observed in the incidence of swallowing difficulty and SIS scores among the three groups. The reason for this difference may be associated with the different survey intervals after thyroidectomy.

The main cause of voice change after thyroidectomy is injury to the recurrent laryngeal nerve (18). However, while voice change may be related to the physiological healing process, others have suggested that smaller incisions, limited dissection, and less trauma to the strap muscles can prevent voice change (18). In a previous study, the RT group achieved better subjective voice outcomes compared to the COT group (18). In our study, however, the incidence of voice change and mean VHI in the ET group were higher than in the RT or COT groups. This discrepancy may be associated with the smaller sample size of the ET group and long learning curve needed for ET.

Our study has several limitations. First, the sizes of the three thyroidectomy approach groups were not equal. In particular, the sample size in the ET group was small. Second, there was a lack of systematic evaluation of postoperative symptoms due to the variable frequency and interval of patient follow-up in the outpatient clinic. In particular, 40 patients (15.7%) had an interval of 6 months or less between their thyroid surgery and survey. Furthermore, the number of survey sessions for postoperative symptoms was only one. In addition, three different nurses conducted the survey. Finally, a large number of total thyroidectomies were included in the COT group, whereas only few were included in the ET and RT groups. Namely, we did not compare postoperative symptoms among the three groups according to hemithyroidectomy or total thyroidectomy and with or without nodal dissection. For clarity, further comparative studies with closely matched patients and procedures are needed.

In conclusion, this is the first study to compare postoperative symptoms after COT, ET, and RT, which were performed by a single surgeon. Our study demonstrated that there were no significant differences in postoperative voice change or swallowing difficulty among the COT, ET, and RT groups, whereas neck pain and discomfort were more common in the ET and RT groups than in the COT group.

## Ethics statement

This study follows the principles expressed in the Declaration of Helsinki. All study participants waived informed consents owing to the retrospective analysis, and the study design was approved by the appropriate ethics review boards.

## Author contributions

DWK: concept and design, review of final manuscript; All authors: acquisition of data, literature review, and refinement of manuscript; HB and DWK: analysis and interpretation of data; TH: manuscript writing.

### Conflict of interest statement

The authors declare that the research was conducted in the absence of any commercial or financial relationships that could be construed as a potential conflict of interest.
